# Language and Vulnerability—A Lacanian Analysis of Respect

**DOI:** 10.3389/fpsyg.2017.02279

**Published:** 2017-12-22

**Authors:** Laurie Laufer, Beatriz Santos

**Affiliations:** Department of Psychoanalytic Studies, Center for Research in Psychoanalysis, Medicine and Society, Paris Diderot University, Sorbonne Paris Cité, Paris, France

**Keywords:** language, vulnerability, symptom, recognition, clinical practice

## Abstract

Lacan's original approach to language expands the reaches of psychoanalysis. Not limited to a set of technical instructions that guide “treatments of the soul,” lacanian psychoanalysis can be seen as a theoretical toolbox whose utility is multidisciplinary. This paper contends that, by establishing a connection between (i) the idea that subjects are produced by language and bear the mark of the unconscious; and (ii) an approach to the production of symptoms that acknowledges the importance of their sense, lacanian theories enlighten discussions on the theme of vulnerability. We claim that Lacan's description of psychoanalysis as an apparatus that respects the person and (foremost) their symptoms generates evidence of the existence of a kind of recognition that takes into account the vulnerability of a given subject without assigning them to a fixed position of victim. This perspective enriches contemporary debates on the relationship between identity and vulnerability.

## Introduction

By referring to contemporary French psychoanalysts interested in the theme of symptoms, the present work examines how psychoanalytical theories on the relationship between language and subjectivity allow for a broader understanding of the concept of vulnerability. A lacanian perspective on respect and its importance to the development of psychoanalytical treatment stems from this discussion on vulnerability.

## Language and reality

In Clarice Lispector's *The passion according to G.H.*, at the end of an introspective quest that culminates in a Kafkaesque encounter with a cockroach, the main character finally understands what language is: “Reality is the raw material, language is the way I go in search of it—and the way I do not find it” (Lispector, 1964/1988). G.H. is a Brazilian middle-class woman who sets out to simply clean a room in her house but finds herself exploring the very origins of human communication. In doing so, she seems to comprehend language as that which allows one to seize the raw material that comes from an external reality (as opposed to psychic reality, in Freud's definition). In other words, G.H is able to experience the discontinuity between the available sensory information (the sense data) and what is captured and organized by our psychic apparatus. G.H's experience allows her to understand that this *capture* cannot happen without language.

As human beings, our very subjectivity is defined by language. As Emile Benveniste puts it, a separation between man on one side and the use of language on the other is not possible: even though we are inclined to imagine a primordial time when a man discovered another one and between the two of them language was worked out little by little, this is not what happened:

“We can never get back to man separated from language and we shall never see him inventing it. We shall never get back to man reduced to himself and exercising his wits to conceive of the existence of another. It is a speaking man whom we find in the world, a man speaking to another man, and language provides the very definition of man” (Benveniste, 1963/1967).

Benveniste insists on the idea that language is much more than an instrument that allows men and women to communicate. The main characteristics that defines language—its immaterial nature, its symbolic functioning, its articulated arrangement and the fact that it has content—set it apart from any instrument created by man: “to speak of an instrument is to put man and nature in opposition. The pick, the arrow, and the wheel are not in nature. They are fabrications. Language is in the nature of man, and he did not fabricate it” (Benveniste, 1963/1967).

This understanding of language as a given that simultaneously precedes and produces the subject proposed by Benveniste is also a main point in Lacan's description of human beings as subjects of language that are subjects *to* language. Throughout his work, Lacan will develop the notion of a subject who is able to talk because he/she *is talked*—that is, because he/she is inscribed in *language as a preexisting structure*. This is a fundamental shift in the understanding of the relationship between human beings and language: once seen as the actor responsible for the performing of acts of speech, the subject becomes, in lacanian theory, the *product* of such acts.

In “Position of the Unconscious” (1960), Lacan develops this idea of a subject subordinated to language through the affirmation that “the effect of language is to introduce the cause into the subject” (Lacan, 1960/2006). For Lacan, the subject is not what he imagines himself to be. We produce an image—an imaginary or specular illusion—of ourselves that protects us against the chaotic movement of our drives. This organized image, known as ego, differs from the subject: the lacanian subject is *the subject of the unconscious*, and is produced by the signifiers of language. The effect of language over the subject thus means that “he ([the subject] is not the cause of himself; he bears within himself the worm of the cause that splits him. *For his cause is the signifier, without which there would be no subject in the real*. But this subject is what the signifier represents, and the latter cannot represent anything except to another signifier: to which the subject who listens is thus reduced” (Lacan, 1960/2006).

The signifier allows the subject to occupy a place among all other beings, but does not encompass the totality of what a subject is. The subject is what the signifier represents; as the famous aphorism goes, the signifier is characterized by the fact that it represents a subject to another signifier (and to another, and to another, in an endless signifying chain, as Lacan will describe it).

## The meaning of symptoms

This understanding of the role played by language in the very constitution of a subject has important implications for the clinical work derived from lacanian theory. One of them is the appreciation of the importance of symptoms to the analytical cure. In his first Seminar, Lacan posits that the symptom initially appears to us as “a trace which will continue not to be understood *(qui restera toujours incomprise)* until the analysis has got quite a long way and we have discovered its meaning (*son sens*)” 1954. This means that, in psychoanalytical theory, the symptom is not simply seen as a manifestation associated to a disease. It is not the indication of a disturbance in the (healthy) condition of a person. Rather, it should be understood as a formation of the unconscious that the analyst should not strive to quickly extinguish since it was carefully (albeit unconsciously) produced by the subject—not unlikely a work of art.

We think of Freud's comparison of symptoms to cultural outputs, and to outputs produced by artists. In 1917, for instance, he mentions the importance of distinguishing the symptoms from the disease of his neurotic patients, and reminds us “that doing away with the symptoms is not necessarily curing the disease. Of course, the only tangible thing left over after the removal of the symptoms is the *capacity to build new symptoms*” (Freud, 1890/1942). This creative capacity may translate into the artist's ability of “turning away from reality” and transferring interests and libido to the elaboration of imaginary wishes. It also works as evidence that symptoms are not to be simply eradicated, but rather taken as an indication that there is work to be done. It is in this sense that Lacan describes the symptom as a trace in his early works: as a mark left by the presence of something that once was at a given place, like footsteps that reveal that someone has stood at a given spot.

What interests us regarding this way of looking at the symptom is the consequences to our approach of the psychoanalytical treatment. What does it mean, to *treat* someone, without getting rid of the symptom but focusing on its *meaning* instead?

French psychoanalyst Sidi Askofaré examines this matter on an article about what he sees as “the revolution of symptom” (Askofaré, 2005)—that is, as the action (by the symptom) of going round in an orbit. The symptom is found at the very beginning of a treatment as the reason why one seeks consultation with an analyst. It is also there at the very end of the analysis, albeit transformed. The trajectory it describes is not one of mere repetition nor of an eternal recurrence of events, but rather a revolution that conjoins a return to and a metamorphosis of events. In the unpublished Seminar from 1976, *L'insu que sait de l'une-bévue s'aile à mourre*, Lacan wonders if, in the end, it would be possible to understand psychoanalysis as synonym to identifying with one's symptom. Not understanding the meaning of the symptom or having it revealed by the analyst, but, as Askofaré puts it, *taking ownership of* the meaning of this symptom: “what is expected from the act of the analyst is that it brings the analysant to take ownership of *(assumer*) the meaning (sexual, phallic, or castration) of his symptoms.”

Askofaré insists on the fact that this ability to assume or undertake the meaning of a symptom radically differs from the mere *understanding* or *treatment* of said symptom. In analysis, what happens to a subject is closer to an ethical experience that Lacan associates with the idea of *respect*. We believe that this specific understanding of respect broadens the use of psychoanalytical theory and contributes for contemporary discussions on vulnerability and politics.

## Respect, recognition, vulnerability

In his very first Seminar, from 1953 to 1954, Lacan studies Freud's articles about psychoanalytical technique. In a lesson concerning the concepts of resistance and defenses, he examines the criticism regarding Freud's supposed “authoritarianism” in relation to his patients—some of Lacan's students describe Freud's handling of the resistance as an act of *conquering* said resistances. Consequently, Freud is seen by these students as someone who is moved by a “strong will for domination.”

But Lacan does not agree with his students' interpretation of Freud's technique. He posits that “if anything constitutes the originality of the analytic treatment, it is rather to have perceived at the beginning, right from the start, *the problematical relation of the subject to himself*. The real find, the discovery, in the sense I explained to you at the beginning of the year, is *to have conjoined this relation with the meaning of the symptoms*” (Lacan, 1953-1954/1988). This means that, rather than acting dominantly, the psychoanalyst works from a position of a certain vulnerability.

Indeed, when Lacan mentions “the problematical relation of the subject to himself,” he is referring to the Freudian notion of *Nebenmensch*, “the fellow human being.” In Freud's work, the (helpful) person capable of removing the distress of the child through a “specific action” also creates—via the same action—dependency and vulnerability. According to Freud,

“Let us suppose that the object which furnishes the perception resembles the subject—a fellow human-being (*nebenmensch*). If so, the theoretical interest taken in it is also explained by the fact that such an object was simultaneously the subject's first satisfying object and further his first hostile object, as well as his sole helping power. For this reason it is in relation to a fellow human-being that a human being learns to recognize” (Freud, 1895/1966).

In other words, it is by being vulnerable and by being exposed to the power and the hostility of another fellow human being that one develops his or her abilities of recognition. A moment of crisis and a critical environment are indeed the very conditions to the development of human's capacity to recognize others. This has a technical consequence that shall bring us back to the lacanian definition of the analyst as someone who gives up knowledge in the same way he/she gives up of his/her ideals.

In fact, Freud described in 1890 a menacing aspect inherent to all situations where *help* is involved (Freud, 1895/1966). Since the forces that work toward helping a subject necessarily impact the “autocratic nature of the personalities of the subjects,” a common reaction in patients is to avoid asking for help of any kind (psychological, medical, or on a social context). The very idea of *being helped* elicits defenses. Consequently, the efficacy of psychoanalytical practice must rely on the fact that it differs from a psychological *aid*. It has to avoid what we could describe, from a lacanian perspective, as the imaginary trap (*le piège imaginaire)* of intersubjectivity. Rather, it should adhere to an unconditional recognition of the symptom of the subject, as well as of the “problematical relationship of the subject to himself” that the symptom imposes.

This unconditional recognition means questioning one's relationship to knowledge. The analyst behaves as a *nebenmensch*, a fellow human who cannot know what the analysant needs. One cannot, as an analyst, assume a position where prescribing attitudes or behaviors is a possibility. Rather, one must give up one's knowledge regarding his/her patients and the illusion of power that comes with it. By doing so, we rend ourselves more vulnerable. But we also move closer to the meaning of the symptoms.

We understand that this is the only way to keep psychoanalysis from either being dissolved into some sort of sentimental psychologisation that fails to take into account the *submission to language* described by Lacan; or into a medical way of thinking that tries to answer to normative ideals regarding treatments. One could describe this position concerning psychoanalysis as *a certain style*, neither *intimate*, nor *extimate* (as Lacan puts it), but *proximate*. As a *practice*, psychoanalysis remains *vulnerable*, situated between two spots, fragile.

In other words, the originality of the analytic treatment is to oppose something very simple to both an inquisitive style of the analysis of resistances and the mere eradication of symptoms: *respect* for the human being and for his or her symptoms. Lacan insists that:

“It is the subject's refusal of this meaning [of the symptom] that poses a problem for him. This meaning must not be revealed to him, it must be *assumed* by him. In this respect, psychoanalysis is a technique which *respects* the person—in the sense in which we understand it today, having realized that it had its price—not only respects it, but cannot function without respecting it” (Lacan, 1953-1954/1988).

From this perspective, *respect* means an idea of care for the other or for oneself that unties itself from a monolithic representation of who this other or this self *should be*. The lacanian understanding of respect allows for an idea of recognition that relies on a more variable (or less fixed) conception of the self.

This point of view seems to relate to the issue of the relationship between identity and vulnerability, as stated on the work of contemporary philosophers such as Judith Butler. In *Precarious Life*, Butler describes how “each of us is constituted politically in part by virtue of the social vulnerability of our bodies—as a site of desire and physical vulnerability.” In other words, the author claims that, from a political perspective, being recognized as a subject implies a feeling of identity that is fundamentally related to an experience of vulnerability:

≪ loss and vulnerability seem to follow from our being *socially constituted bodies*, attached to others, at risk of losing those attachments, exposed to others, at risk of violence by virtue of that exposure ≫ (Butler, 2004 p. 20).

Throughout her work, Butler has described how the act of being called a name paradoxically limits a subject *and* allows himself or herself to exist. When one receives a name—woman, man, transgender, Brazilian, heterosexual, gay, etc.—, one receives the possibility to exist socially at the same time that he or she is supposed to abide to the characteristics that allow for such a naming. Different characteristics lead to different designations and, consequently, to different degrees of vulnerability.

≪ one speaks, and one speaks for another, to another, and yet there is no way to collapse the distinction between the Other and oneself. When we say ≪ we ≫, we do nothing more than designate this very problematic. We do not solve it. And perharps it is, and it ought to be, insoluble. This disposition of ourselves outside ourselves seems to follow from bodily life, from its vulnerability and its exposure ≫ (Butler, 2004 p. 25).

The trouble with (any) identity, according to Judith Butler, lies in its variability and its diversity—≪ I am a woman. Which woman ? ≫. But this very trouble, this difficulty, is what allows us to understand how recognition cannot rely on an opaque, fixed, or monolithic representation of a subject.

## Conclusion

These theoretical developments invite us to rethink what is at stake in the relationship between recognition and vulnerability. French psychoanalyst Jean Allouch argues that the psychoanalyst establishes a relationship to “variety as such”(le *divers comme tel*) which implies refraining from assigning a subject to a predefined clinical entity—or to a predefined name. In Allouch's words, this means that “oriented by variety, the psychoanalyst is bound to welcome anyone, and to do so by restraining from any identificatory action or thought” (Allouch, 2014). This means assuming a delicate position where one is perpetually thinking the subject without references to a knowledge of preexisting categories. And this ability to recognize variety without reducing it to rigid categories stems from this respectful attitude toward language, in the sense suggested by Lacan. Such an attitude is essential to the successful conduction of psychoanalytical treatments, and it may also enlighten discussions on vulnerability rising from other fields.

## Author contributions

All authors listed have made a substantial, direct and intellectual contribution to the work, and approved it for publication.

### Conflict of interest statement

The authors declare that the research was conducted in the absence of any commercial or financial relationships that could be construed as a potential conflict of interest. The handling Editor declared a shared affiliation, though no other collaboration, with the authors.
